# Erlotinib Inhibits Growth of a Patient-Derived Chordoma Xenograft

**DOI:** 10.1371/journal.pone.0078895

**Published:** 2013-11-15

**Authors:** I-Mei Siu, Jacob Ruzevick, Qi Zhao, Nick Connis, Yuchen Jiao, Chetan Bettegowda, Xuewei Xia, Peter C. Burger, Christine L. Hann, Gary L. Gallia

**Affiliations:** 1 Department of Neurosurgery, Johns Hopkins University School of Medicine, Baltimore, Maryland, United States of America; 2 Johns Hopkins University School of Medicine, Baltimore, Maryland, United States of America; 3 Ludwig Collaborative Laboratory, Johns Hopkins University School of Medicine, Baltimore, Maryland, United States of America; 4 Department of Oncology, Johns Hopkins University School of Medicine, Baltimore, Maryland, United States of America; 5 Ludwig Center for Cancer Genetics, Johns Hopkins University School of Medicine, Baltimore, Maryland, United States of America; 6 Department of Neurosurgery, Affiliated Hospital of Gulin Medical College, Guilin, China; 7 Department of Pathology, Johns Hopkins University School of Medicine, Baltimore, Maryland, United States of America; Utrecht University, Netherlands

## Abstract

Chordomas are rare primary bone tumors that occur along the neuraxis. Primary treatment is surgery, often followed by radiotherapy. Treatment options for patients with recurrence are limited and, notably, there are no FDA approved therapeutic agents. Development of therapeutic options has been limited by the paucity of preclinical model systems. We have established and previously reported the initial characterization of the first patient-derived chordoma xenograft model. In this study, we further characterize this model and demonstrate that it continues to resemble the original patient tumor histologically and immunohistochemically, maintains nuclear expression of brachyury, and is highly concordant with the original patient tumor by whole genome genotyping. Pathway analysis of this xenograft demonstrates activation of epidermal growth factor receptor (EGFR). *In vitro* studies demonstrate that two small molecule inhibitors of EGFR, erlotinib and gefitinib, inhibit proliferation of the chordoma cell line U-CH 1. We further demonstrate that erlotinib significantly inhibits chordoma growth *in vivo*. Evaluation of tumors post-treatment reveals that erlotinib reduces phosphorylation of EGFR. This is the first demonstration of antitumor activity in a patient-derived chordoma xenograft model and these findings support further evaluation of EGFR inhibitors in this disease.

## Introduction

Chordomas are rare primary bone tumors that arise in the cranial base, mobile spine, and sacrococcygeal region. Current treatment includes surgical resection and postoperative radiotherapy [1]. Despite such treatment, local recurrence occurs in the majority of patients [2], metastasis may occur in at least 20% of patients [2], and the median survival for patients is 6-7 years [3]. Notably, there are no FDA approved agents for patients with chordoma. These facts highlight the need for identification of new treatment options.

A major limitation of therapeutic development for chordoma is the lack of preclinical models. As reviewed recently by Tentler et al. [4], there has been an increase in the use of patient-derived xenografts (PDX) which better recapitulate the heterogeneity, genomics, and drug responsiveness of primary human tumors. We previously described the establishment of the first chordoma PDX [5] and, in this study, we further characterize this model and demonstrate *in vivo* efficacy of EGFR inhibition.

## Materials and Methods

### Cell lines

The human chordoma cell line U-CH 1 [6] was kindly provided by the Chordoma Foundation (Durham, NC) and grown on plates coated with sterile 0.1% gelatin (Sigma, St. Louis, MO) in a media formulation of 4 parts IMDM (Invitrogen, Carlsbad, CA):1 part RPMI (Sigma) with 10% fetal bovine serum (Gemini Biosciences, West Sacramento, CA) and 1X penicillin/streptomycin (Invitrogen). The lung carcinoma cell line A549 was obtained from ATCC (Rockville, MD) and grown as recommended.

### Ethics statement

 This study was carried out in strict accordance with the recommendations in the Guide for the Care and Use of Laboratory Animals of the National Institutes of Health. The protocol was approved by the Johns Hopkins Animal Care and Use Committee (Protocol #MO10M89). All surgery was performed under an approved rodent ketamine/xylazine anesthesia cocktail and all efforts were made to minimize suffering. 

### Xenografts

Mice were housed in standard facilities, given free access to Baltimore City water and chow, and monitored frequently for signs of tumor growth. The chordoma PDX (JHH-2009-011) was propagated and maintained as previously described [5]. Briefly, tumor was harvested, minced with razor blades, mixed 1:1 with reduced-growth factor Matrigel (BD Biosciences, San Jose, CA), and subcutaneously injected into the flanks of 5-6 week old female athymic nude mice (NCI, Bethesda, MD). 

### Histopathology and Immunohistochemistry

Samples of the original patient tumor from which the PDX line was generated and xenografts were fixed in 10% buffered formalin and embedded in paraffin. Five micron sections were deparaffinized and stained with hematoxylin and eosin (H & E) or antibodies specific for brachyury (1:50, Santa Cruz Biotechnology, Santa Cruz, CA), cytokeratin AE1/AE3 (predilute, Ventana/Roche, Tuscon, AZ), EGFR (EGFR PharmDx Kit, Dako, Carpinteria, CA), EMA (predilute, Ventana/Roche), Ki-67 (predilute, Ventana/Roche), and S100 (predilute, Ventana/Roche). For brachyury immunohistochemistry, citrate buffer (BioGenex, San Ramon, CA) was used for antigen retrieval. Antibody detection was achieved using a biotinylated secondary antibody and horseradish peroxidase-conjugated streptavidin (Ventana/Roche) for EMA, cytokeratin AE1/AE3, Ki-67, and S100. Horseradish peroxidase-conjugated anti-goat polymer (Dako) was used to detect EGFR and brachyury staining. Immunostaining was visualized with 3’, 3’ diaminobenzidine (Dako). 

### Receptor Tyrosine Kinase (RTK) arrays

The Human RTK Phosphorylation Antibody Array 1 and Human EGFR Phosphorylation Antibody Array 1 (RayBiotech, Norcross, GA) were used according to the manufacturer’s recommendations. The RTK array was probed with 200 ug/ml of xenograft extract (passage 5). The EGFR arrays were probed using 400 ug/ml of xenograft extracts from animals treated with vehicle or erlotinib.

### Flow cytometry analysis

Xenografts were harvested, mechanically dissociated to obtain single cell suspensions, rinsed with PBS, and passed twice through 70 µM cell strainers. One million cells were incubated with 20 µL of PE-conjugated mouse anti-human EGFR antibody (BD Biosciences) or PE-conjugated mouse isotype control antibody (IgG2b κ, BD Biosciences) for 30 minutes at RT. A549 was used as a positive control. Cells were pelleted, washed, and analyzed on a FACSCalibur II (BD Biosciences). 

### SNP analysis

The Illumina Human 1M BeadChip, HumanOmni2.5-4v1 (Illumina, San Diego, CA), contains more than 1.2 million common single nucleotide polymorphism (SNP) markers and was utilized to analyze copy number variations (CNV) in genomic DNA isolated from the original patient tumor and passages 1, 2, 3, and 4 of the PDX. Neoplastic cellularity was greater than 80% in each of the samples analyzed. All SNP positions were mapped to the hg19 (NCBI Build 37/hg19) version of the human reference genome. Log R ratios and B-allele frequencies were calculated and outputted by GenomeStudio (2010v2, Illumina). The overall genotype calling rate reached above 98% for all samples. Genes affected by CNV in regions larger than 100,000 base pairs were detected and reported by KaryoStudio (v1.4, Illumina).  Visualization of copy number changes in a genomic interval above 100,000 base pairs as well as copy number gain or loss at the chromosome arm level was generated by KaryoStudio based on B-allele frequency and log R ratio. 

### Sequencing

 Primers used to amplify all 28 exons of *EGFR* have been previously described [7], except those used to amplify exon 3 for which the following primers were used: (forward) M13F- ACTGGGCGTCCTAGGGCTC and (reverse) GCCTTGGCATCCCAGCCTC. The M13F sequencing primer used was GTAAAACGACGGCCAGT. Genomic DNA was extracted from xenograft tumor using the DNeasy kit (Qiagen, Valencia, CA) following the manufacturer’s instructions. Genomic DNA was extracted from peripheral blood leukocytes, obtained from the patient under a Johns Hopkins Institutional Review Board-approved protocol, using Puregene Blood Kit chemistry on an Autopure LS automated DNA purification instrument according to the recommendations of the manufacturer (Qiagen). Both tumor and normal DNA were diluted to a concentration of 30 ng/ul and the following PCR mix was utilized to amplify each exon. PCR was performed in 5 μl reactions containing 1× PCR Buffer (67 mM Tris-HCl, pH 8.8, 6.7 mM MgCl_2_, 16.6 mM NH_4_SO_4_, 10 mM 2-mercaptoethanol), 1 mM dNTPs (Invitrogen), 1 μM forward and 1 μM reverse primers, 6% DMSO, 2 mM ATP, 0.25 U Platinum *Taq* (Invitrogen) and 3 ng DNA. Reactions were carried out in a 384-well ABI 9700 thermocycler (Applied Biosystems) using a touchdown PCR protocol: 1 cycle of 96°C for 2 min; 3 cycles of 96°C for 10 sec, 64°C for 10 sec, 70°C for 30 sec; 3 cycles of 96°C for 10 sec, 61°C for 10 sec, 70°C for 30 sec; 3 cycles of 96°C for 10 sec, 58°C for 10 sec, 70°C for 30 sec; 41 cycles of 96°C for 10 sec, 57°C for 10 sec, 70°C for 30 sec; 1 cycle of 70°C for 5 min. Sanger Sequencing was performed on the samples by Genewiz (South Plainfield, NJ). Using data from the normal DNA as the reference, the DNA sequencing results were analyzed using Mutation Surveyor (State College, PA).

### *In vitro* studies

Erlotinib (Tarceva™) and gefitinib (Iressa™) tablets, kindly provided by Dr. Nisana Namwat (Khon Kaen University, Khon Kaen, Thailand), were crushed and then dissolved in DMSO for *in vitro* studies. Between 2,000 - 4,000 U-CH 1 cells were plated in 96-well plates in 200 μL of U-CH1 media and treated the following day with vehicle (0.25% DMSO) or various concentrations of erlotinib and gefitinib ranging from 2.5 nM to 25 uM for 48 h. Proliferation assays were performed using the Cell Titer 96® AQueous One Solution Proliferation Assay according to the recommendations of the manufacturer (Promega, Madison, WI) with minor modifications. Briefly, 20 μL of CellTiter96 AQueous One reagent was added to each well after removing 100 μL of media from each well. Plates were incubated for 3-5 hours at 37°C. Absorbance was measured using the Victor^3^ microplate reader (Perkin-Elmer, Walthan, MA). Background absorbance (media alone) was subtracted from each treatment absorbance value and percent inhibition calculated based on DMSO control. Experiments were repeated at least three times with a minimum of 5 replicates in each experiment. 

### *In vivo* efficacy studies

Xenografts were propagated as described above. Erlotinib tablets were crushed and dissolved in PBS for *in vivo* studies. When tumors reached an average size between 200 and 250 mm^3^ (approximately 35 days post-tumor implantation), daily treatment with oral gavage of control vehicle (PBS) or erlotinib (50 mg/kg) commenced. Animals were weighed and tumors measured twice weekly with calipers. When tumors reached 2,000 mm^3^, animals were euthanized and tumors harvested. Tumor volume was calculated by the formula for an ellipsoid: pi/6 x length x width x height [8]. Tumor growth curves were plotted using GraphPad Prism (GraphPad, La Jolla, CA). Statistical differences between growth curves were calculated using the nonparametric Mann-Whitney-Wilcoxon test.

## Results

### The chordoma PDX maintains the histological, immunohistochemical and genomic profile of the original patient tumor

**Figure 1:**
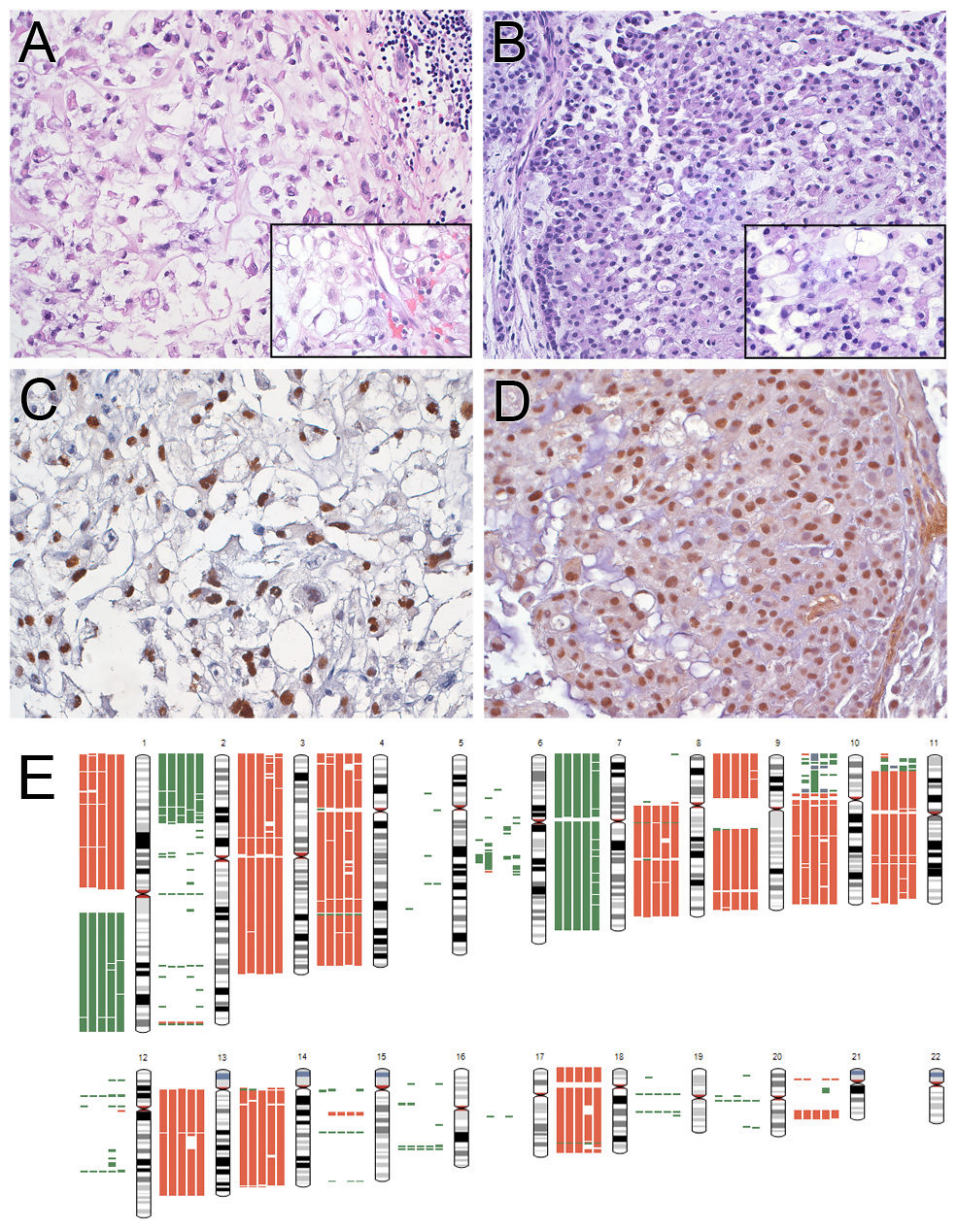
The chordoma PDX maintains the histological and immunohistochemical profile of the original patient tumor. A and B. The patient’s clival tumor (A) and PDX, passage 8, (B) retained the classic chordoma features, including physaliphorous cells (inset). Both the patient's tumor (C) and PDX, passage 8 (D), were immunoreactive for brachyury. Magnifications: A and B, 100x; inset 260x; C and D, 160x. E. Karyotype of chordoma genomes. Estimation of copy number gains and losses in the original patient sample (left most bar) and PDX passages 1, 2, 3 and 4 (left to right). Green bar = gain; red bar = loss. Chromosome 7 has one copy number gain including the *EGFR* locus.

We previously reported the establishment and initial characterization of a chordoma PDX [5]. This lineage has been growing as primary xenografts for the past 40 months and is currently in passage 11. The time between passages has remained stable at approximately 100 days. This chordoma PDX continues to resemble the original patient tumor histologically (Figure 1A and B). Both the original tumor and xenograft have the classic compact architecture of chordoma, with lobules of cells variably separated by thin fibrous septa with sprinklings of chronic inflammatory cells. Tumor cells were generally cohesive, but there were loose areas with individual cells and prominent faintly basophilic extracellular matrix. Typical of chordoma, cords of cells with an epithelioid appearance due to sharp cytoplasmic borders were present. A sizeable minority of cells contained vacuoles. There were generally one or two vacuoles per cell, but many were present in some cells and multivacuolated, i.e. physaliphorous, cells were also present. Nuclear pleomorphism was restrained and only rare mitoses were identified. The PDX also maintained the immunohistochemical profile of the original patient tumor. Both tumors were diffusely positive for EMA, cytokeratin AE1/AE3, and S100 (Figure S1). Almost all of the nuclei in the original patient tumor and PDX were positive for brachyury, a marker for chordoma (Figure 1C and D). 

CNV were assessed in the original patient tumor and passages 1, 2, 3, and 4 of the PDX. All four xenograft passages shared almost identical CNV with the original patient sample. Mostly, detected regions were single copy loss or single copy gain of one chromosome arm with more losses than gains (Figure 1E). Complete loss of two alleles or two copy number gain could have occurred in a few smaller segments. No high copy number amplification was identified. Detailed CNV analysis is listed in Table S1. We previously performed CNV analysis on the patient’s sample and two xenograft passages in which we characterized genome deletions and focal amplifications [5]. Although a different array platform and cutoff parameters were used, the results from the current study are consistent with our previous data. 

### EGFR is activated in the chordoma PDX

**Figure 2:**
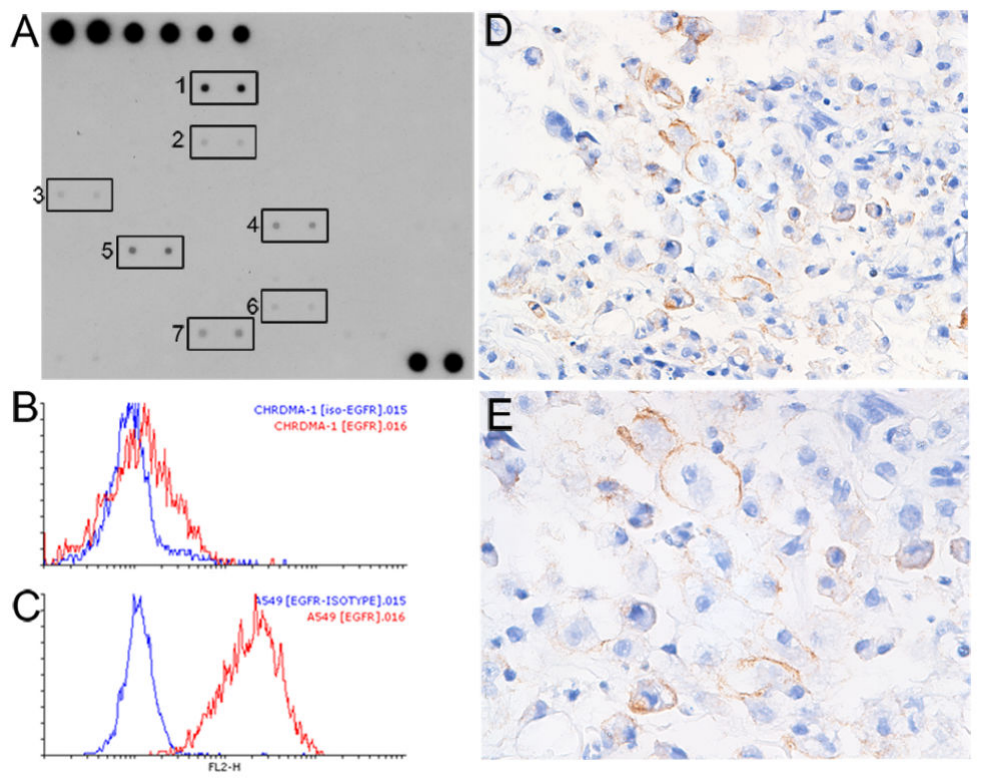
EGFR is activated in the chordoma PDX. A. RTK phosphorylation array of the PDX demonstrated EGFR activation (box 1). Other activated kinases include EphB4 (2), Fgr (3), JAK2 (4), Lyn (5), SRMS (6), and TNK1 (7). The first six columns of the first row and last two columns of the last row contain positive controls. B and C. Analytical flow cytometry of the chordoma PDX (B) and A549 (C) for EGFR. Red line: anti-EGFR antibody; blue line: isotype control antibody. D and E. EGFR staining of the original patient tumor demonstrated surface staining of scattered tumor cells. Magnifications: D, 160x; E, 260x.

To further characterize this PDX, a RTK phosphorylation array was screened. Several kinases were noted to be activated and, of the 71 kinases on this array, EGFR was the most activated (Figure 2A). Based on this observation, we analyzed the xenograft for EGFR expression by flow cytometry and found a low level of EGFR expression on the tumor cells (Figure 2B and C). To determine if this was similar to the level of expression in the original tumor, immunohistochemistry for EGFR was performed on the original patient specimen. Surface membrane staining for EGFR was present on a minority of cells (Figure 2D and E), consistent with flow cytometric findings in the xenograft. 

As *EGFR* polysomy and amplification have been reported in chordoma [9–13], we examined *EGFR* copy number. Analysis of the original patient tumor and xenografts revealed that although a copy number gain was detected for the whole chromosome 7, where *EGFR* is located, the *EGFR* locus was not significantly amplified (data not shown). To determine if activating mutations were present, all 28 exons of *EGFR* were sequenced. No mutations were identified. Taken together, these results demonstrate that the original patient tumor and xenograft express EGFR and, of those examined, EGFR is the most activated kinase in the xenograft. Moreover, this activation is neither due to amplification nor activating mutation.

### EGFR inhibition reduces chordoma growth *in vitro*

**Figure 3:**
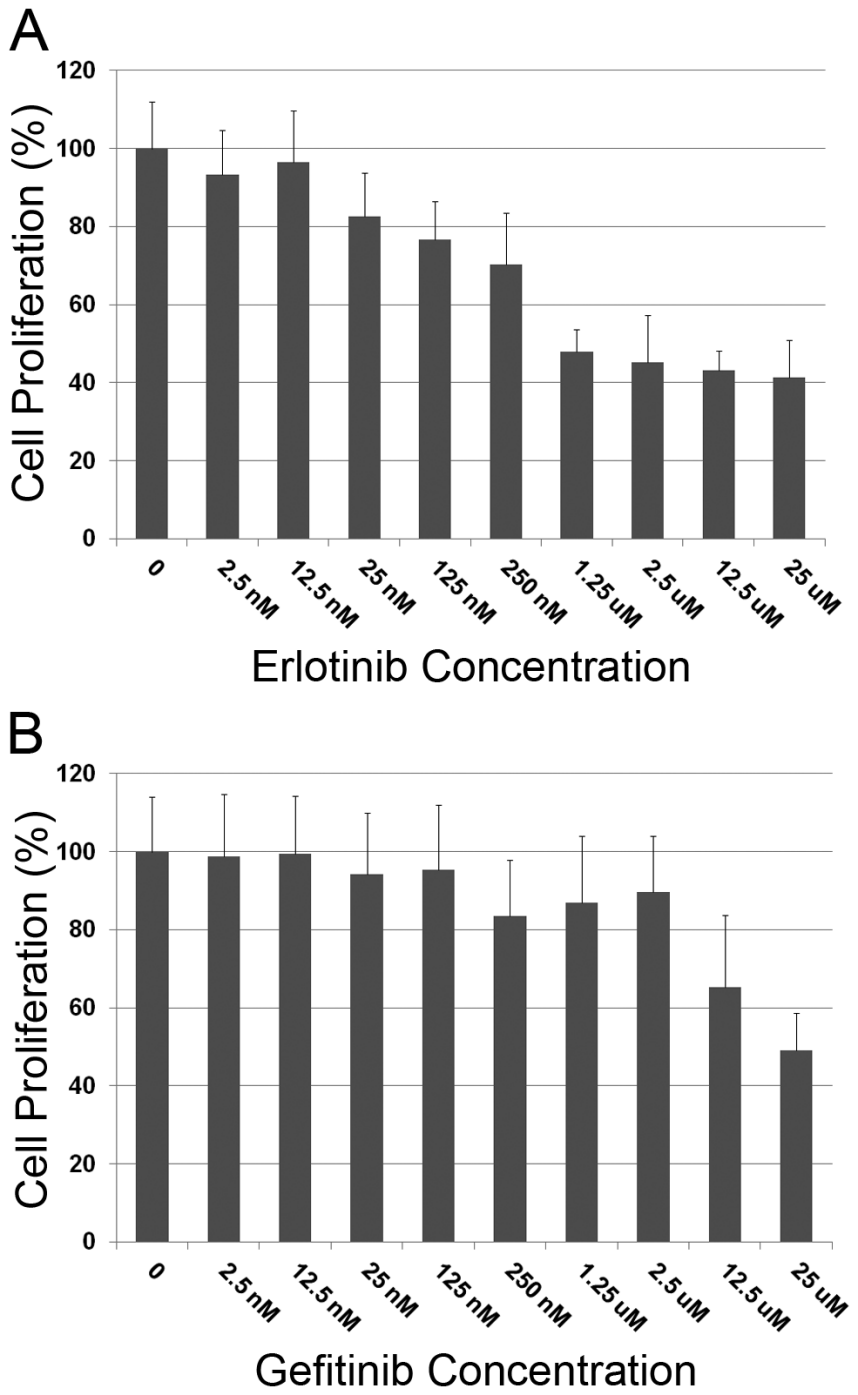
Erlotinib and gefitinib inhibit growth of U-CH1 *in* *vitro*. Proliferation assays were performed following treatment of U-CH1 cells with control and increasing concentrations of erlotinib (A) and gefitinib (B). Data shown is mean relative cell proliferation (percent of control) + standard deviation. Experiment was repeated at least 3 times with quantitatively similar results.

Given activation of the EGFR pathway in the chordoma PDX, we next evaluated the efficacy of small molecule EGFR inhibitors against a validated chordoma cell line U-CH 1, which has also been shown to have activated EGFR [11]. In this series of experiments, U-CH1 cells were treated with vehicle (DMSO) and increasing concentrations of erlotinib and gefitinib ranging from 2.5 nM to 25 uM. As 1% DMSO significantly inhibited growth of U-CH1 (data not shown), the final concentration of DMSO in these experiments was 0.25%. Both erlotinib and gefitinib inhibited proliferation of U-CH1 in a dose-dependent fashion (Figure 3). Erlotinib was more efficacious and, for this reason, was selected for *in vivo* studies.

### Erlotinib inhibits chordoma xenograft growth *in vivo*

**Figure 4:**
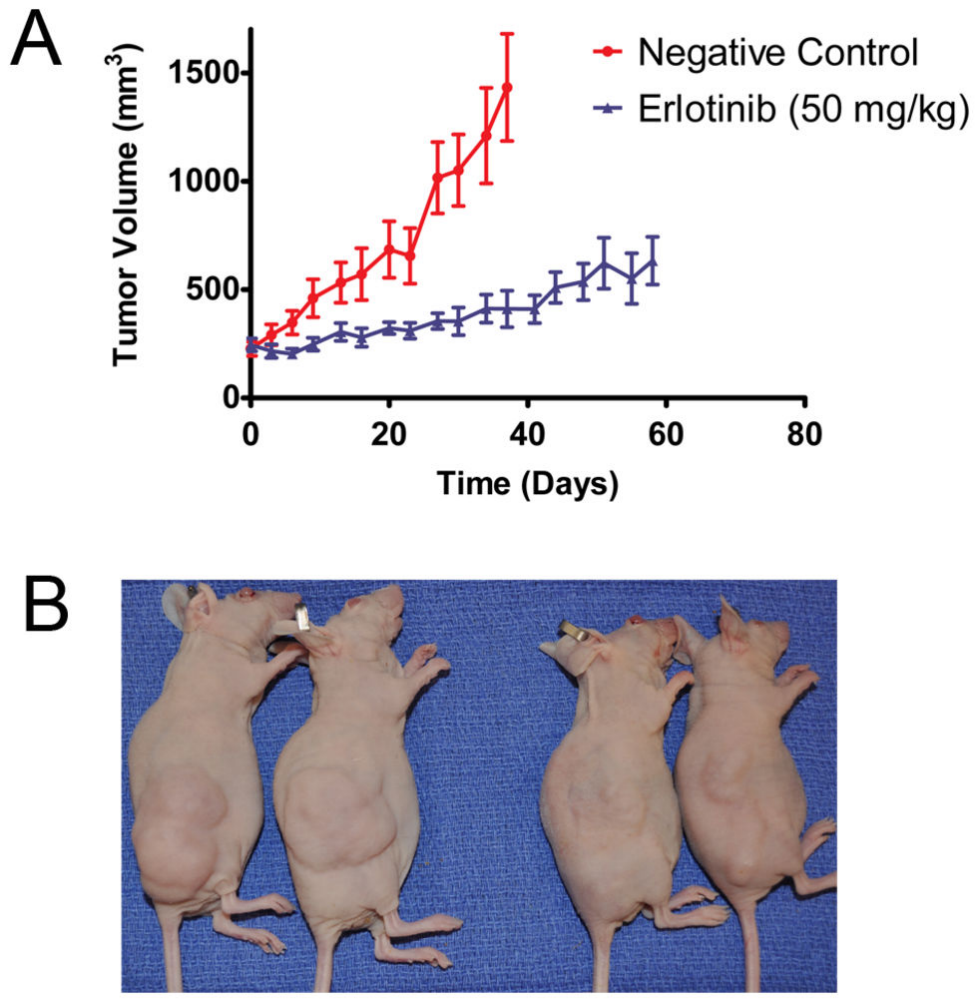
Erlotinib inhibits growth of the chordoma PDX. A. Growth curves of animals treated with vehicle (red line) or erlotinib (50 mg/kg, blue line) (p = 0.002). The growth curve of vehicle treated animals was censored at 37 days as tumors in two animals in this group reached 2,000 mm^3^ at this time point and were euthanized. B. Representative mice bearing flank xenografts treated with vehicle (left) and erlotinib (right).

To ascertain the effect of EGFR inhibition *in vivo*, we examined the efficacy of erlotinib in our chordoma PDX. Xenografts were treated with vehicle control (n = 7) or erlotinib (n = 7). After 37 days of treatment, tumors in two animals in the vehicle-treated group reached 2,000 mm^3^; at this timepoint, the average tumor volume for the control group was 1433 mm^3^ while the average tumor volume for the erlotinib-treated group was 411.3 mm^3^ (Figure 4). At treatment day 58, animals in the erlotinib group were euthanized and tumors were harvested. The average tumor volume at this time point was 633.3 mm^3^, with none reaching 2,000 mm^3^ (Figure 4). The growth curves were statistically significantly different (p= 0.002). This experiment was repeated in a separate set of animals with quantitatively similar results.

**Figure 5:**
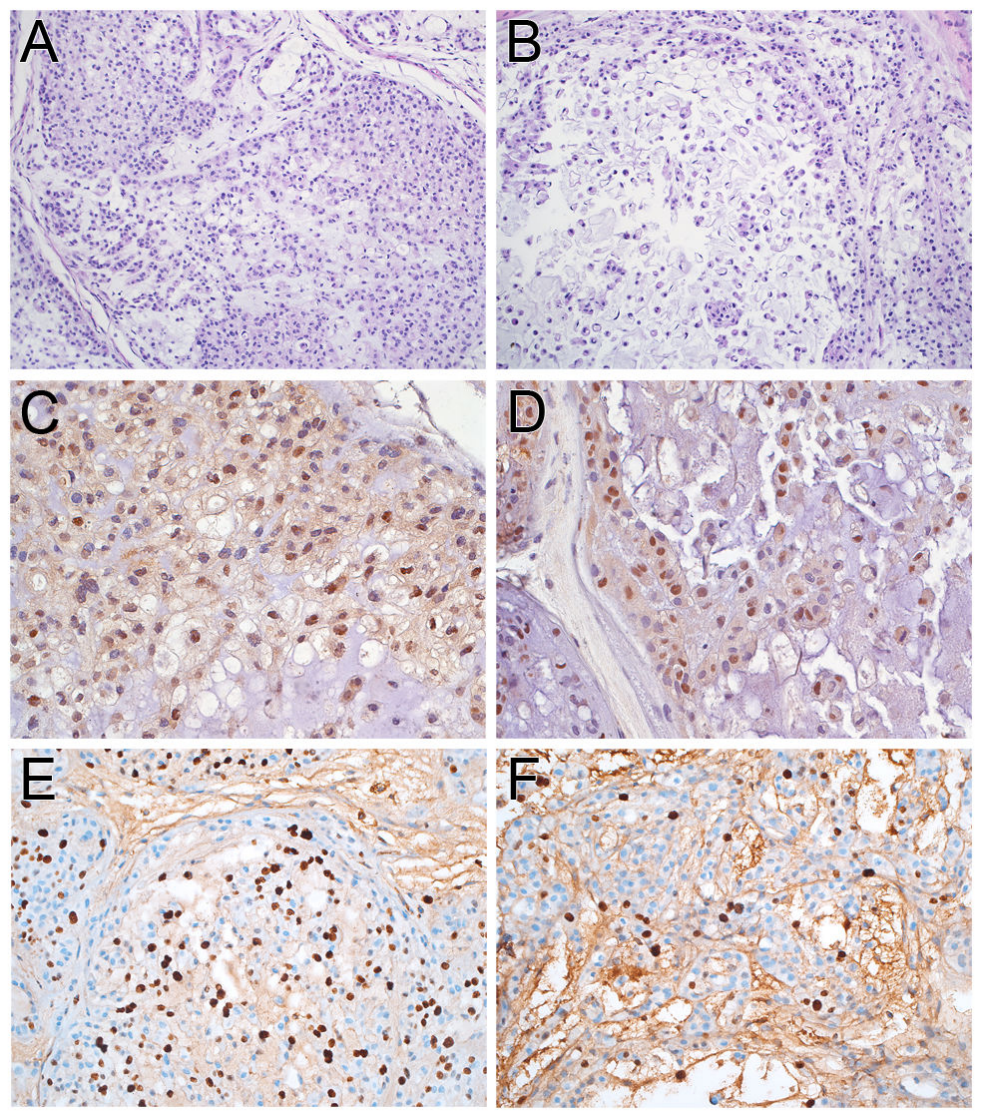
Pathological changes in the PDX following treatment with erlotinib. A. The control xenograft was composed largely of compact tissue with only a minority of loose, less cellular tumor. B. Loose, discohesive, less cellular areas were more common in erlotinib-treated xenografts. C and D. Nuclei in the control (C) and erlotinib (D) treated xenografts were immunoreactive for brachyury. E. Control treated xenografts had a brisk Ki-67 index. F. Ki-67 indices were generally lower in erlotinib-treated xenografts. Magnifications: A and B, 64X; C and D, 160X; E and F, 100x.

**Figure 6:**
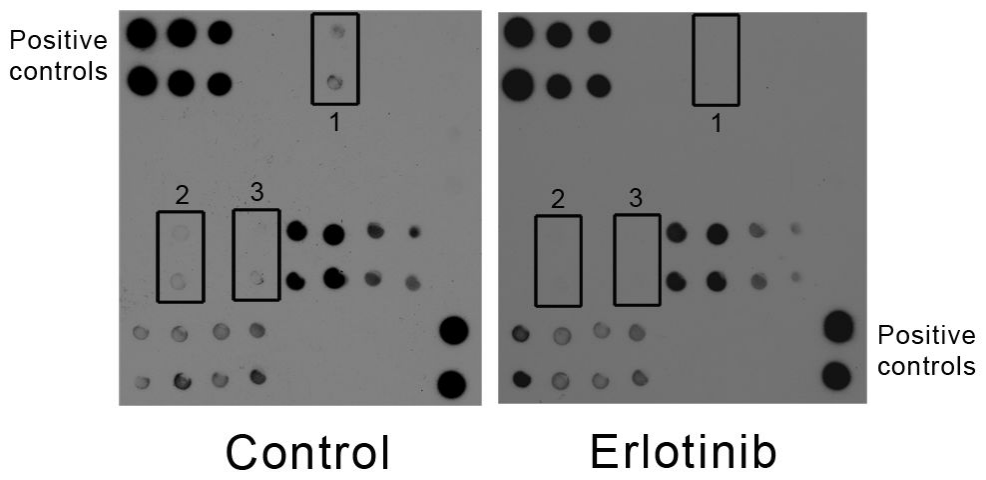
Phosphorylation of EGFR is reduced following treatment with erlotinib. Representative EGFR phosphorylation arrays from control (left) and erlotinib treated tumors (right) demonstrated reduced phosphorylation of the Tyr845 site of EGFR (box 1) following treatment with erlotinib. The Erb2 phosphorylation sites Tyr 1112 and Tyr 1248 were also reduced (boxes 2 and 3). The first 3 columns of the first two rows and the last column of the last two rows contain positive controls.

Examination of H & E-stained sections revealed the control tumors had the classic architecture of chordoma (Figure 5A) as described above. The erlotinib-treated xenografts were histologically similar to the control-treated counterparts with the exception that the former generally had more prominent loose centrilobular areas with cell-cell dehiscence (Figure 5B). There was no change in brachyury staining with almost all tumor cell nuclei positive (Figure 5 C and D). Ki-67 positive nuclei were present throughout the control tumors, but were often more numerous at the periphery of the nodules where the tumor was more cohesive. The index was estimated in these areas to be 20-30%. In the erlotinib-treated xenografts, Ki-67 positive nuclei were present throughout the tumor and also were often more numerous at the periphery of the nodules where the tumor was more cohesive. While there was some overlap with the control grafts, the index varied from 10-20%. Using an array that detects phosphorylation of numerous sites in EGFR family members, we found that erlotinib treatment resulted in a reduction in phosphorylation of Tyr845 on EGFR; we also noted a decrease in phosphorylation of two tyrosine residues on Erb2 (Figure 6). 

## Discussion

In earlier work, we established and characterized the first and only reported chordoma PDX to date [5]. In the current study, we further characterized this model and demonstrate continued fidelity of the xenograft to the original patient tumor histopathologically, immunohistochemically, and genomically by CNV analysis. Further analysis demonstrated that EGFR was the most activated kinase in a panel of 71 RTKs and that the EGFR inhibitors erlotinib and gefitinib significantly inhibited proliferation of U-CH1 *in vitro*. Extending these findings to *in vivo* studies, we also demonstrated that erlotinib significantly inhibited growth of the chordoma PDX.

 There are several previous reports evaluating EGFR expression in chordoma [9–15]. These immunohistochemistry-based studies reported that between 32 and 100% of the chordoma samples examined were positive for EGFR. Activation of EGFR has also been investigated in several studies; these studies reported between 43 and 100% of chordomas express phosphorylated EGFR [10–12,15]. In the largest study evaluating EGFR in chordoma, EGFR expression was reported in 79 out of 114 (69%) chordomas and 57 out of 115 (51%) samples expressed phosphorylated EGFR [11]. 

EGFR polysomy has been reported in a variable number of chordoma samples (17-52%) by FISH [9–12]. In the largest of these studies, Shalaby et al. demonstrated that close to 40% of 147 chordomas had high *EGFR* copy number [11]. In our case, analysis of the patient’s tumor and xenografts demonstrated that, although a copy number gain was detected for chromosome 7, the *EGFR* locus was not significantly amplified. These data suggest that EGFR activation in chordomas is not solely due to increased copy number. 

Another mechanism of EGFR activation is by gene mutation/deletion. Perhaps the best model for EGFR mutation-driven tumorigenesis is in non-small cell lung cancer where activating mutations within exons 18-21 of *EGFR* have been reported and predict for response to EGFR inhibitors [16,17]. Dewaele et al. [10], Tamborini et al. [12], and Shalaby et al. [11] examined 13, 22, and 62 chordomas, respectively, for activating mutations in exons 18-21 of *EFGR* and no mutations were identified. As *EGFR* mutations have been reported in exons other than 18-21 in other malignancies [18], we sequenced all 28 exons of *EGFR* in our chordoma PDX; no mutations were identified. Activation of EGFR may also be resultant from an autocrine/paracine loop with overexpression of its ligands. Although not explored in our xenograft, EGF and TGFα were reported to be highly expressed in 22 chordoma samples (100%) in a previous study [12].

Our array analysis demonstrated that EGFR is significantly activated in the chordoma PDX, consistent with other recent reports on chordoma samples [10–12]. To functionally investigate the role of EGFR in chordoma, Shalaby et al. demonstrated that the EGFR inhibitor tyrphostin AG 1478 decreased proliferation of a verified chordoma cell line U-CH 1 [11]. We similarly found that erlotinib and gefitinib inhibited U-CH1 proliferation in a dose-dependent manner. Our *in vivo* studies demonstrated that erlotinib treatment resulted in a significant decrease in growth of our chordoma PDX. As expression of EGFR appears restricted to a minority of cells in the PDX, the explanation for the pronounced *in vivo* effect of erlotinib may be multi-factorial. Specifically, although erlotinib is considered a specific EGFR inhibitor, it has been reported to inhibit other kinases including ErbB2 [19] and Src family members [20] in other tumor types. Interestingly, we noted a decrease in phosphorylation of some tyrosine phosphorylation sites on ErbB2 following erlotinib treatment. In addition, the Src kinases, Fgr and Lyn, were activated in our chordoma PDX. It is possible that inhibition of these, or other activated pathways, may contribute to the observed *in vivo* effect of erlotinib. Furthermore, we postulate that EGFR inhibition may have a more dramatic effect on tumors with higher EGFR expression. Additional testing in other chordoma PDXs, as they become available, will be important to evaluate the general applicability of these findings in chordoma.

To date, there have been few prospective clinical studies in chordoma [21,22]. Though EGFR activation has frequently been observed in chordomas [10–12,15], EGFR inhibitors have rarely been used clinically in this disease. To the best of our knowledge, four case reports have described the use of EFGR inhibitors in patients with chordoma, specifically with a combination of cetuximab and gefitinib [23,24] or erlotinib alone [25,26]. In all four cases, treatment with EGFR inhibitors led a response. Notably, two patients who received multiple prior treatments for recurrent chordoma including resection, radiotherapy, and imatinib, had responses > 11 months to erlotinib therapy [25,26]. Recently, a study by Stacchiotti et al. was published on an exploratory phase II study involving 18 patients with metastatic or locally advanced EGFR-positive chordoma treated with lapatinib, a tyrosine kinase inhibitor active against both EGFR and HER2/neu [27]; sixteen of the 18 patients had prior therapy with imatinib. In this study, six patients (33.3%) had partial response and seven patients (38.9%) had stable disease as assessed by Choi criteria. Median progression-free survival by Choi criteria was 6 months and by RECIST 8 months. The clinical benefit rate was 22% and one patient was progression-free at greater than 12 months. Four of 10 evaluable patients (40%) had a decrease in PET scan uptake [27]. 

In this paper, we demonstrate EGFR is a highly activated kinase in a patient-derived chordoma xenograft, erlotinib and gefitinib inhibit U-CH1 proliferation *in vitro*, and erlotinib inhibits growth of chordoma *in vivo*. These results, together with the limited clinical experience, demonstrate efficacy of EGFR inhibition in chordoma and support additional investigation of anti-EGFR therapy in this disease. 

## Supporting Information

Figure S1 **The original patient tumor (**A**, **C** and **E**) and chordoma PDX (**B**, **D**, and **F**) were immunoreactive for EMA (**A** and **B**), cytokeratin AE1/3 (**C** and **D**) and S100 (**E** and **F**).** Magnification in all panels was 160X.
(TIF)

Table S1 **Detailed copy number variations in the original patient tumor and passages 1, 2, 3, and 4 of the PDX predicted by Illumina KaryoStudio based on the HumanOmni2.5 SNP array.**
(XLSX)
