# Supplementary figures and images for: Erlotinib Inhibits Growth of a Patient-Derived Chordoma Xenograft
Source: PLoS One. 2013 Nov 15;8(11):e78895. doi: 10.1371/journal.pone.0078895 (PMC3829812; doi:10.1371/journal.pone.0078895)

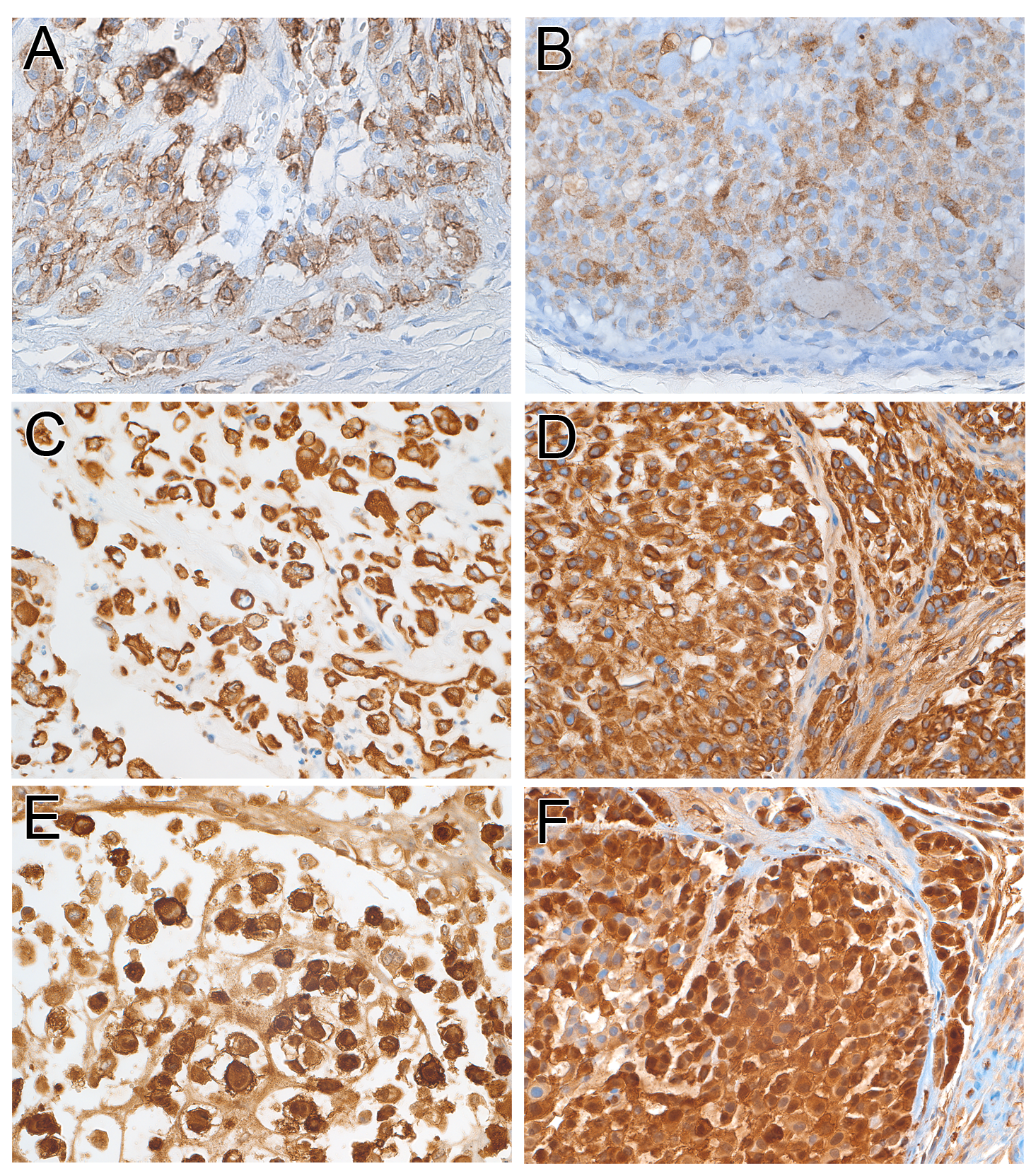

Supplement: Figure S1 — The original patient tumor (A, C and E) and chordoma PDX (B, D, and F) were immunoreactive for EMA (A and B), cytokeratin AE1/3 (C and D) and S100 (E and F). Magnification in all panels was 160X. (TIF) [file pone.0078895.s001.tif]
